# Medical practitioner’s knowledge on dengue management and clinical practices in Bhutan

**DOI:** 10.1371/journal.pone.0254369

**Published:** 2021-07-16

**Authors:** Tsheten Tsheten, Archie C. A. Clements, Darren J. Gray, Kinley Gyeltshen, Kinley Wangdi

**Affiliations:** 1 Department of Global Health, Research School of Population Health, Australian National University, Canberra, Australia; 2 Royal Centre for Disease Control, Ministry of Health, Thimphu, Bhutan; 3 Faculty of Health Sciences, Curtin University, Perth, Australia; 4 Telethon Kids Institute, Nedlands, Australia; 5 Phuntsholing General Hospital, Phuntsholing, Bhutan; MAHSA University, MALAYSIA

## Abstract

**Background:**

Dengue has emerged as a major public health problem in Bhutan, with increasing incidence and widening geographic spread over recent years. This study aimed to investigate the knowledge and clinical management of dengue among medical practitioners in Bhutan.

**Methods:**

We administered a survey questionnaire to all practitioners currently registered under the Bhutan Medical and Health Council. The questionnaire contained items on four domains including transmission, clinical course and presentation, diagnosis and management, and surveillance and prevention of dengue. Participants were able to respond using an online Qualtrics survey, with the invitation and link distributed via email.

**Results:**

A total of 97 respondents were included in the study (response rate: 12.7%), of which 61.86% were Health Assistants/Clinical Officers (HAs/COs) and 38.14% were medical doctors. The afternoon feeding behaviour of *Aedes* mosquito was correctly identified by only 24.7% of the respondents, and ~66.0% of them failed to identify lethargy as a warning sign for severe dengue. Knowledge on diagnosis using NS1 antigen and the clinical significance of elevated haematocrit for initial fluid replacement was strikingly low at 47.4% and 27.8% respectively. Despite dengue being a nationally notifiable disease, ~60% of respondents were not knowledgeable on the timing and type of cases to be reported. Respondent’s median score was higher for the surveillance and reporting domain, followed by their knowledge on transmission of dengue. Statistically significant factors associated with higher knowledge included respondents being a medical doctor, working in a hospital and experience of having diagnosed dengue.

**Conclusion:**

The study revealed major gaps on knowledge and clinical management practices related to dengue in Bhutan. Physicians and health workers working in Basic Health Units need training and regular supervision to improve their knowledge on the care of dengue patients.

## Introduction

Dengue, one of the most rapidly spreading vector-borne viral diseases, is found in tropical and sub-tropical regions around the world [1]. Before 1970, only nine countries experienced severe dengue epidemics [2]. However, dengue is now endemic in over 140 countries in Africa, the Americas, the Eastern Mediterranean and Asia [3]. The 2002 World Health Assembly Resolution WHA55.17 urged greater commitment to dengue control among member states, owing to its rapidly increasing public health importance. Subsequently, the IHR 1969 was revised in 2005 (WHA58.3) to include dengue among the public health emergencies of international concern (PHEIC) [4].

Reducing dengue morbidity and mortality requires an organized process of early detection, accurate classification, notification, treatment and referral when necessary [5]. Early detection and selecting the most appropriate treatment for dengue is of paramount importance to prevent the progression of the disease to severe dengue and subsequent case fatalities [6]. As a result of appropriate clinical management, case fatalities due to dengue have been significantly reduced from 10–20% to less than 1% in many countries over recent years [7]. Activities such as triaging and management decisions at the primary and secondary care levels are critical in reducing the number of unnecessary hospitalizations and deaths [5]. Timely notification of dengue allows rapid initiation of public health interventions, thereby reducing the transmission of dengue virus in the community [6].

Due to the non-specific clinical presentation, complex case definitions and a lack of routine laboratory diagnosis, dengue is often misdiagnosed, leading to poor clinical management and underreporting [8, 9]. In countries where dengue is imported by travellers, both diagnosis and treatment are delayed due to a lack of clinical suspicion of the disease [10]. Knowledge of dengue in such settings can be poor, with healthcare workers wrongly identifying *anopheles* mosquitoes as the vectors of dengue (15%) [11], use of paracetamol for preventing dengue [12], and prescribing antiviral and antimalarial drugs for treating dengue [13]. Whilst nearly all clinicians in a study in Texas demonstrated awareness that avoidance of mosquito bites is necessary to prevent dengue, only 33% knew that febrile dengue patients should take precautions to avoid mosquito bites to prevent dengue virus transmission to household members [14]. Similarly, in Singapore, only a small proportion of physicians (29.5%) reported performing an early diagnostic test for every suspected dengue cases [15]. A study in Taiwan had found poor knowledge of important clinical characteristics of dengue [6]. All these variations necessitate the need to understand the local characteristics of dengue management practices to develop appropriate strategies to improve population health.

**Fig 1:**
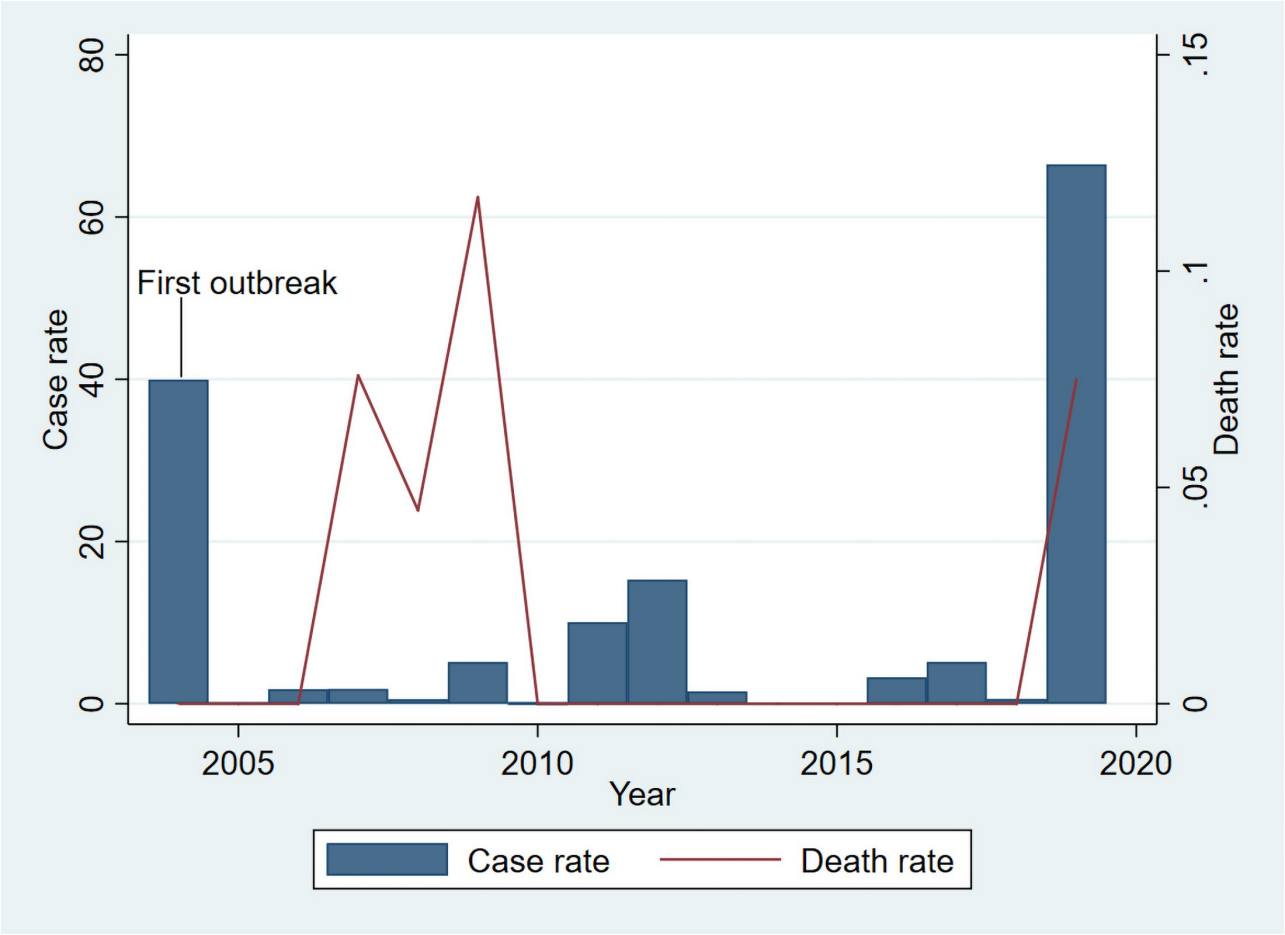
Dengue incidence and death rate (per 10,000) in Bhutan, 2004–2019 (source, Annual Health Bulletin, Royal Centre for Disease Control, and WHO-SEARO).

Bhutan experienced its worst ever dengue epidemic in 2019. The Royal Centre for Disease Control under the Ministry of Health recorded 5,935 dengue cases during the 2019 epidemic, which is more than all the combined cases reported in previous years. Cases were reported from 19 of the 20 districts and six deaths were reported (Fig 1). The current study aims to characterise dengue-related knowledge and clinical management practices among medical practitioners (MP) in Bhutan in the year following the 2019 outbreak.

## Materials and methods

### Study setting

Bhutan is a small landlocked country measuring 38,394 square kilometres, nestled in the Eastern Himalayas between India in the south, east and west, and China in the north. The right to free access to essential health services is mandated by the constitution, and currently, the expenditure for healthcare services including referrals to third countries is borne by the government of Bhutan. Healthcare services are delivered through 32 hospitals (one-national, two regional and the remainder district general hospitals) and 208 Primary Health Care (PHC) or Basic Health Units (BHUs—grade I and II) [16]. Primary care is delivered in each of these health centres, but medical technologies and healthcare services become more advanced and specialized from BHUs to district general hospitals, to regional hospitals and the national referral hospital. Over 903 MPs are currently registered by the Bhutan Medical and Health Council (BMHC). This includes 92 medical specialists (MD), 186 general duty medical officers (GDMO with a Bachelor of Medicine/Bachelor of Surgery), 21 clinical officers (COs) and 604 Health Assistants (HAs) [16]. HAs undergo two years in-country training for primary care based on a whole-of-society approach that includes health promotion, disease prevention, diagnosis, treatment, rehabilitation and palliative care. These HAs undertake an additional one-year diploma in clinical management to become COs. Although doctors and HA/COs work together in all health facilities, HA/COs predominantly work at BHUs.

### Study design

We conducted a self-administered online survey among MPs in Bhutan from November to December 2019. This cross-sectional study sought to understand the MP’s knowledge and management practices in relation to dengue.

We used Qualtrics (Qualtrics, Provo, UT) to administer the survey questionnaire. Each respondent was sent an invitation message in their email to complete the survey questionnaire, with a URL link embedded within the message. Once the respondents voluntarily agreed to participate in the study, they then navigated to the survey questionnaire. To increase our response rate, as explained by Cook et al. [17], we sent two reminders on days, 14 and 28 after sending out the initial email.

### Respondent recruitment

**Fig 2:**
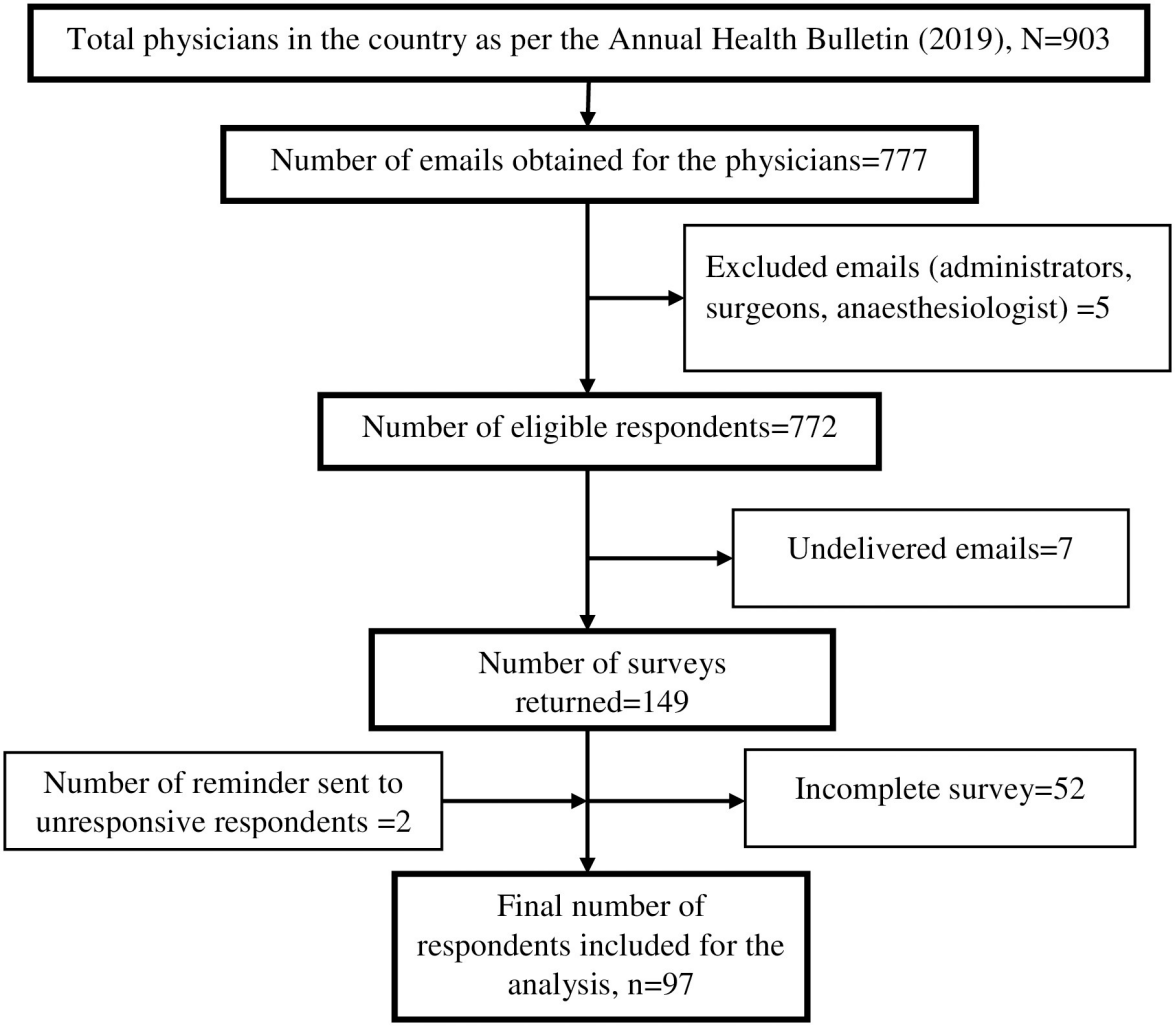
Study population recruitment for assessing knowledge on dengue management among physicians in Bhutan, 2019–2020.

Eligible respondents comprised of any practitioner practising in areas involved in diagnosis and management of dengue patients, including general medicine, paediatrics, emergency medicine, intensive care and laboratory medicine, at any level of the health system. Of the 903 MPs registered by the BMHC, email addresses were obtained for 777 of them. Five were excluded as they didn’t work clinically in areas relevant to dengue (including health service managers, surgeons and anaesthesiologists), thus creating a potential respondent group of 772 MPs (Fig 2). Survey questionnaires were emailed to all respondents from December 2019 to January 2020. Email addresses were collected from the district health services. The district health managers contacted target respondents via telephone and email to encourage participation in the survey. Reminder telephone calls were also made by managers to enhance participation of the respondents. No incentives or compensations were offered to the respondents.

### Survey questionnaire

Development of the study tool was informed by a review of the literature related to clinical practices for dengue care and past surveys on dengue management [11, 12, 14, 18, 19], and the tool was evaluated by three local experts on dengue. The tool was further modified after feedback from a pilot study which was conducted in Gomtu Hospital, Samtse district. Respondents from the pilot study were excluded in the final survey and analysis. Demographic questions included district, region, age, sex, highest qualification, number of years in service, whether they had diagnosed dengue and their specialty. The main questionnaire consisted of 25 multiple choice questions, which included the following domains: 1) Transmission; 2) Clinical course and presentation; 3) Diagnosis and management; 4) Surveillance and prevention of dengue (S1 Appendix). Since English is the official medium of pedagogy in both schools and medical institutes in the country, the questionnaire was designed in English and not translated into Dzongkha (the national language).

### Data analysis

The response rate was calculated by dividing the total number of surveys returned by the total number of invitations (772). Characteristics of the respondents were described using categorical variables and presented as frequencies and proportions. For simplicity, national, regional and district general hospitals were grouped as “hospital”, while BHU grade I and II were grouped as “BHU”. Correct responses to each question were summarized under separate domains, using frequencies and proportions. A choropleth map was developed in ArcGIS version 10.5 (ESRI, Redlands, CA) for visualizing the proportion of respondents (%) who participated in the survey [20].

To compare knowledge across respondents with different characteristics, a cumulative knowledge score was calculated as an aggregate of all the knowledge-based questions separately for each domain. A score of one was given for the right answer. For questions with multiple-choice questions, respondents received a score of one for every correct answer. Normalization was performed to standardize domain scores into values that ranged from 0 to 1 by dividing the respondent’s total score for each domain by the total domain score. Boxplots were created for each domain using the normalized scores. Since the distributions were non-normal as determined by a histogram, knowledge scores of all domains were compared across categories of respondent characteristics using non-parametric tests; i.e. the Man-Whitney U test and the Kruskal-Wallis test. All data analyses were carried out using Stata statistical software (version 15.1; Stata Corp, College Station, Texas, USA) [21].

### Ethical clearance

The study was reviewed and approved by the Research Ethics Board of Health (REBH), Ministry of Health, Bhutan (2019/081) and the Human Research Ethics Committee (HREC), Australian National University (ANU), Australia (2019/808).

## Results

### Response rate

Of the 772 respondents to whom the invitation was sent, we received notification that seven emails were not delivered. In addition, 52 respondents responded but did not complete the survey questionnaire, and were thus excluded from the response rate and subsequent analysis. Among those respondents who did not complete the survey, 14 were from the BHUs, two were from the national referral hospital and seven were from district hospitals. However, 27 of the respondents did not complete the health centre details. In terms of roles, 17 were HAs/COs, five were medical doctors and the rest did not provide any information. We got a response from a total of 97 respondents who had completed the questionnaire, yielding a total response rate of 12.7% (97/765) (Fig 2).

### Characteristics of the respondents

**Fig 3:**
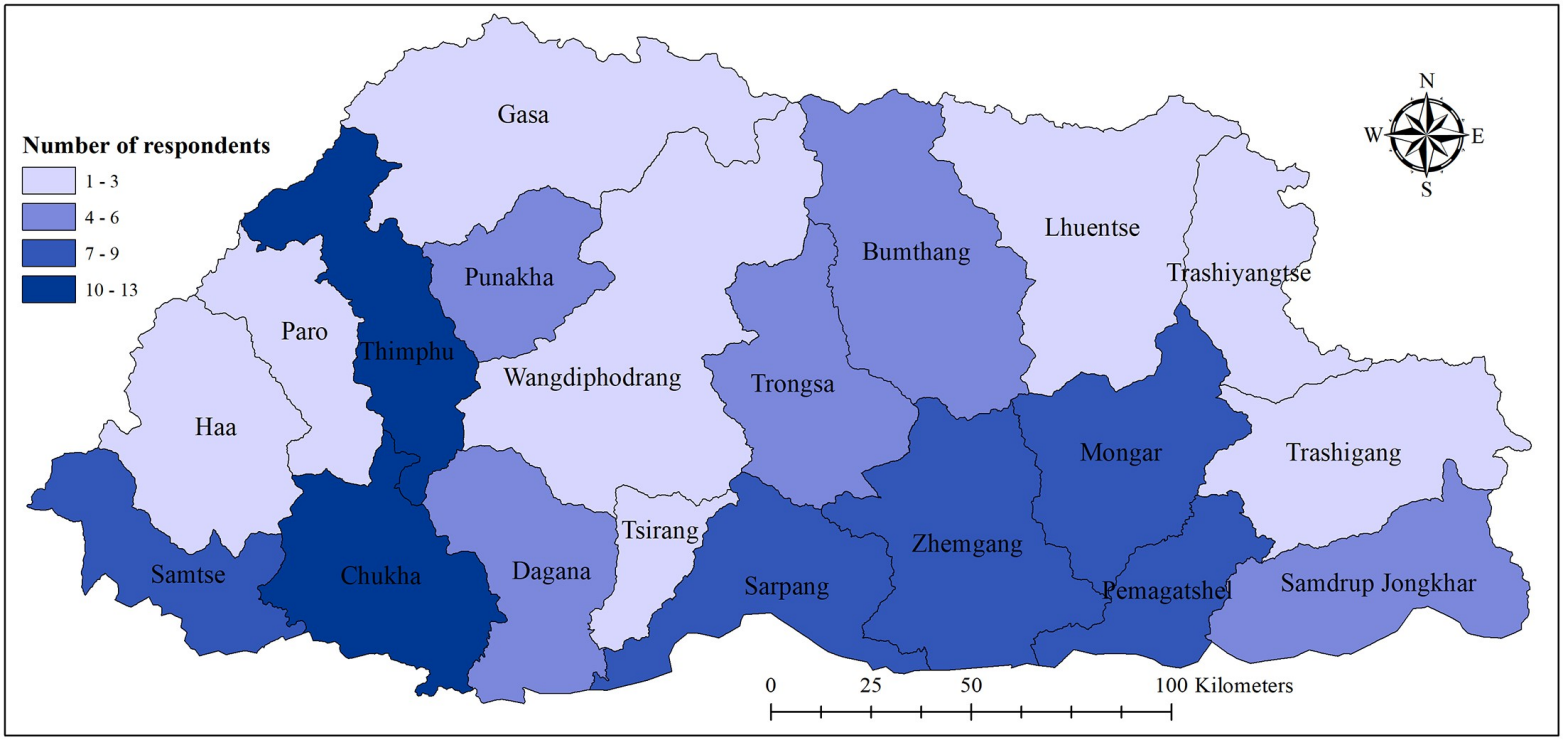
District-wise number of respondents included in the study, Bhutan, 2019–2020.

Chukha district had the highest frequency of respondents (n = 13) (Fig 3). The age group with the largest number of respondents was <30 years (44.3% of the total) and most respondents were male (72.2%). There was a greater number of respondents who were HAs/COs (61.9%) than medical doctors (38.1%). Respondents from all health centres participated in the survey, including one from a private diagnostic centre. General medicine/practice was the most commonly reported specialty (90.7%) followed by emergency services (3.1%). More than half of the respondents had personally diagnosed dengue in a patient (55.7%) (Table 1).

**Table 1:** Characteristics of the study respondents.

Variables	Response selected	n	%
Age (years)	<= 29	43	44.3
	30–39	34	35.1
	>= 40	20	20.6
Sex	Male	70	72.2
	Female	27	27.8
Qualification	Certificate/Diploma	48	49.5
	BPH/M.Sc	5	5.2
	MBBS	36	37.1
	MD+PhD	8	8.3
Medical role	HAs/COs	60	61.9
	Medical doctors	37	38.1
Health centre level	Hospital	44	45.4
	BHU	52	53.6
	Private diagnostic	1	1.0
Medical speciality	General medicine	88	90.7
	Paediatric	2	2.1
	Emergency	3	3.1
	Obstetrics/gynaecology	2	2.1
	Laboratory/Radiology	2	2.1
Medical experience (years)	<= 5	50	51.6
	> 5	47	48.5
Ever diagnosed	Yes	54	55.7
	No	43	44.3

BPH: Bachelor of Public Health. HA: Health Assistant. CO: Clinical Officer. BHU: Basic Health Unit.

### Knowledge on the transmission of dengue (domain 1)

The majority of respondents (89.7%) correctly identified *Aedes* mosquitoes as the vector of dengue. Whilst more than half of the respondents reported mornings and evenings as times favourable for feeding behaviour of vector mosquitoes, only 24.7% correctly identified afternoons as a favourable feeding time. Only 16.7% reported that when a mosquito bites a dengue patient, it might become infected and then infect the next person it encounters during feeding time. About one-third of the respondents were aware of different dengue serotypes circulating in the country (Table 2).

**Table 2:** Knowledge on the transmission of dengue (domain 1), Bhutan, 2019–2020.

Question	Response	n (%)
1. How is dengue transmitted?	1. By the bite of *Aedes* mosquitoes	87 (89.7)
2. When do *Aedes* mosquitoes bite a person?	2.1. Morning	60 (61.9)
	2.2 Afternoon	24 (24.7)
	2.3. Evening, until dark	73 (75.3)
3. When a mosquito bites a dengue patient, which of the following is true?	3.1 Mosquito might get infected and can infect next person during feeding time	16 (16.5)
	3.2. There is no infection of the mosquito if DENV is cleared from the human body	86 (88.7)
4. So far, which of the DENV serotypes are identified in Bhutan?	4.1. DENV-1	69 (71.1)
	4.2. DENV-2	61 (62.9)
	4.3. DENV-3	26 (26.8)

### Clinical course/presentation of dengue (domain 2)

Most of the study respondents (88.7%) were knowledgeable about the duration of the incubation period of infection with dengue virus. While more than half of the respondents correctly indicated options for identifying a patient with dengue at the time of presentation, only 45.4% would ask patients about vomiting, abdominal pain, lethargy and bleeding. Persistent vomiting, severe abdominal pain and bleeding were correctly identified as warning signs for severe dengue by 54.6%, 55.7% and 83.5% respectively. Lethargy was identified as a sign of severe dengue by only 34.0% of respondents. A notable lack of knowledge was observed on the classification of dengue based on the given clinical scenario; only 27.8% were able to correctly identify the right option of “dengue with warning signs” (Table 3).

**Table 3:** Knowledge of clinical course/presentation of dengue (domain 2), Bhutan, 2019–2020.

Question	Correct response/s	n (%)
1. How long does it take to develop symptoms of dengue?	1. Between 3–7 days after mosquito bite	86 (88.7)
2. How should a clinician identify patients with dengue at the time of presentation?	2.1. Take fever history	55 (56.7)
	2.2. Perform complete blood count	53 (54.6)
	2.3. Ask about headache, retro-orbital pain, body-aches and rash	75 (77.3)
	2.4. Ask about vomiting, abdominal pain, lethargy and bleeding	44 (45.4)
3. Identify the warning signs of dengue:	3.1. Persistent vomiting	53 (54.6)
	3.2. Lethargy	33 (34.0)
	3.3. Bleeding	81 (83.5)
	3.4. Severe abdominal pain	54 (55.7)
4. A patient presents to you with a history of fever, headache, joint pain, vomiting, mucosal bleeding, lethargy and liver enlargement. This patient is classified as:	4. Dengue with warning signs	27 (27.8)
5. Severe dengue is considered if the patient from an area of dengue risk presenting with 2–7 days of fever has any of the following clinical manifestations:	5.1. Evidence of plasma leakage	55 (56.7)
	5.2. Significant bleeding	63 (65.0)
	5.3. Lethargy or restlessness	46 (47.4)
	5.4. Persistent vomiting or acute abdominal pain	50 (51.6)
	5.5. Severe organ impairment	46 (47.4)
6. Which is the best early indicator of shock?	6. Tachycardia in the absence of fever or delayed capillary refill	43 (44.3)
7. When does clinically significant plasma leakage usually develop?	7. Between 3–7 days around the time of defervescence	69 (71.1)
8. Thrombocytopenia is expected to develop:	8. Following progressive leukopenia, around the time of defervescence	45 (46.4)

### Diagnosis and management of dengue (domain 3)

Respondents less frequently identified NS1 antigen for early diagnosis (47.4%) than anti-dengue IgM antibodies (62.9%). Chest X-ray with a lateral decubitus view for pleural effusion and haematocrit for diagnosing plasma leakage were reported as parts of the diagnostic approach for dengue by 27.8% and 56.7% respectively. Only 21.7% of the respondents correctly reported that patients with pre-existing co-morbidities need to be closely monitored before discharging them. Of the indications identified for administering IV crystalloids such as Normal Saline or Ringer’s Lactate to dengue patients, hypotension and tachycardia, or low urine output, were identified by 78.4% and 50.5% respectively. Surprisingly, only 27.8% correctly identified high haematocrit as an indication for initial fluid replacement. For a given scenario of a six-year-old boy with fever of a few days duration, distended painful abdomen and lethargy, 66.0% of the respondents reported that they would order a dengue test and admit the patient for 24 hours of observation (Table 4).

**Table 4:** Knowledge on diagnosis and management of dengue (domain 3), Bhutan, 2019–2020.

Question	Correct response/s	n (%)
1. What is true about testing dengue virus?	1.1. Anti-dengue IgM antibodies are first detectable in most patients on days 3–5 after illness onset	61 (62.9)
	1.2. NS1 Ag appears as early as day 1 & can be used for early diagnosis	46 (47.4)
2. How is significant plasma leakage detected in a suspected dengue patient?	2.1. Increasing hematocrit above 20% of baseline	55 (56.7)
	2.2. Chest X-ray with lateral decubitus view to look for pleural effusion	27 (27.8)
3. The best course of action for a 6-year old boy with fever of a few days duration, distended painful abdomen and lethargy is to:	3. Order a dengue lab test and admit the patient for 24 hours of observation	64 (66.0)
4. Select the therapies that you would use for managing dengue fever	4.1. Paracetamol	80 (82.5)
	4.2. Oral rehydration	74 (76.3)
5. Which of the following are the correct criteria for sending a suspected dengue patient home?	5.1. Passing urine at least once every 6 hours	36 (37.1)
	5.2. Does not have major co-morbidities	21 (21.7)
	5.3. Does not have any warning signs	82 (84.5)
	5.4. No hemoconcentration	39 (40.2)
6. Under what circumstances do you tell a patient with suspected dengue to return to the clinic?	6.1. Persistent vomiting	69 (71.1)
	6.2. Drowsiness or lethargy	64 (66.0)
	6.3. Hematemesis	65 (67.0)
7. When should IV crystalloids (like Ringer’s Lactate or Normal Saline) be given to suspected dengue patients?	7.1. Hypotension (as initial fluid replacement)	76 (78.4)
	7.2. High hematocrit (as initial fluid replacement)	27 (27.8)
	7.3. Tachycardia, delayed capillary refill, or low urine output (as initial IV fluid therapy)	49 (50.5)
8. When should a blood transfusion be given to patients with suspected dengue?	8.1 Significant clinical bleeding	54 (55.7)
	8.2. Low hematocrit and persistent shock after a trial of IV crystalloids and/or colloids	41 (42.3)

### Surveillance and prevention of dengue (domain 4)

The majority of the respondents were knowledgeable about dengue being a nationally notifiable disease (80.4%). However, only 47.4% knew that suspected or confirmed cases have to be reported every week. More than 80.0% of respondents were able to identify both the variables (i.e., age and case type) included in surveillance reporting. About 95.0% of respondents were able to identify prevention messages such as using mosquito repellent and wearing long sleeve pants and shirts to prevent dengue infection (Table 5).

**Table 5:** Surveillance reporting and prevention of dengue (domain 4), Bhutan, 2019–2020.

Question	Correct response/s	n (%)
1. Which of the following are true about the reporting of dengue in Bhutan?	1.1. Dengue is a notifiable disease	78 (80.4)
	1.2 All suspected or confirmed dengue cases should be reported every week	46 (47.4)
	1.3. Severe dengue should be reported immediately after detection	57 (58.8)
2. Variables required to report dengue cases as per the national surveillance are:	2.1. Age	78 (80.4)
	2.2. Case type (whether death occurred or not)	82 (84.5)
3. Dengue vaccination is recommended for people aged > = 9 years in areas with:	3. >= 70% prevalence of dengue	57 (58.8)
4. What prevention message should be given to patient to prevent dengue infection?	4.1. Use of mosquito repellent, and wearing long sleeve shirts and pants	92 (94.8)
	4.2. Taking precautions to avoid the bite of mosquitoes if any member of the household is infected with dengue	68 (70.1)

### Comparisons of domains by respondent’s characteristics

**Fig 4:**
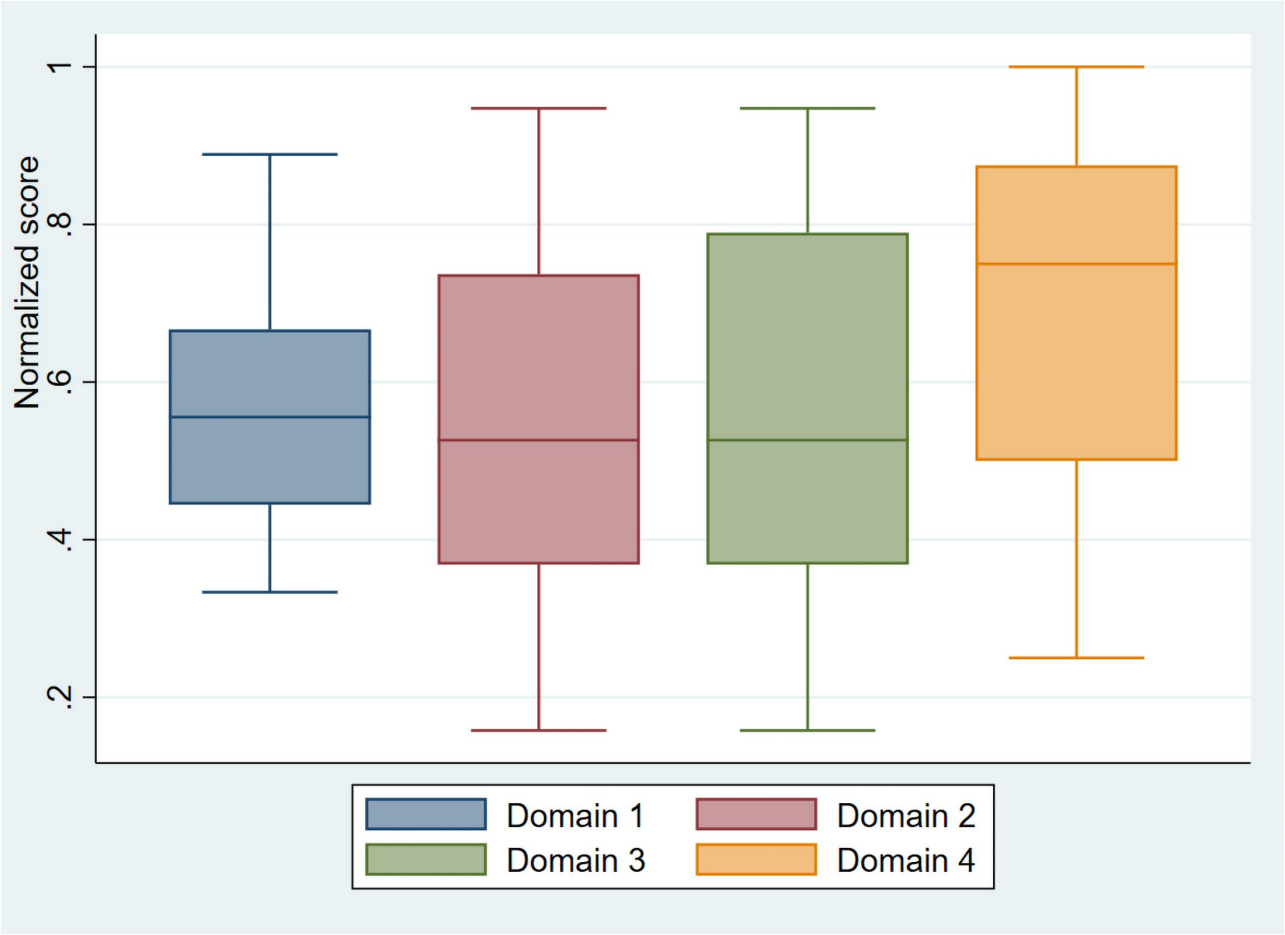
Boxplot showing respondent scores for four domains on knowledge of dengue management in Bhutan, 2019–2020. Domain 1: Transmission; domain 2: Clinical course; domain 3: Diagnosis and treatment; domain 4: Surveillance and prevention.

The cumulative median score was 32 (IQR: 22–39) out of a possible score of 54 for all the four domains. Boxplots demonstrated higher median scores for domain 4 followed by domain 1. Domain 2 and 3 had the same median score with slightly lower upper quartile scores for domain 2 (Fig 4). Being a medical doctor and having diagnosed dengue were significantly associated with a higher median score across all domains (p-value<0.05). Those working in the hospital had a significantly better score for the first three domains (p-value<0.05). Performance of the respondents did not vary by age group, sex or medical experience for any of the domains (S1 Table).

## Discussion

MPs play a crucial role in early detection and timely management of dengue patients, and notification of dengue cases to national authorities. Having a high level of accurate knowledge on dengue management amongst these professionals can save the lives of patients and prevent other healthy people from becoming infected. The current study revealed major gaps in the knowledge of MPs on transmission, clinical presentation, diagnosis and treatment, and prevention of dengue in Bhutan. Knowledge varied according to the type of profession, healthcare setting and past experience in managing dengue cases. To our knowledge, this is the first study of health practitioner’s knowledge and practices with regards to dengue in the WHO-South East Asia Region (WHO-SEAR).

In this study, poor knowledge on transmission of dengue was evident from the respondent’s inability to recognize the feeding time of dengue mosquitoes. Similar findings have been documented by Huang et al. in Taiwan, where, only 14.4% of respondents correctly identified the feeding behaviour of dengue mosquitoes [22]. Having accurate knowledge on the behaviour of dengue mosquitoes will help MPs to impart appropriate health education [22], which remains one of the cornerstones of preventing dengue.

Respondents had low knowledge of the signs that lead to dengue shock and thrombocytopenia. Such findings were also reported in Puerto Rico, where only 29.0% of the respondents correctly identified early signs of shock, and 48.0% identified severe abdominal pain and persistent vomiting as warning signs of severe dengue [23]. This could be due to a lack of training on the recognition of warning signs and case classification of dengue as per the updated WHO guidelines. Identification of warning signs of dengue and indications that lead to shock is critical for managing dengue [1]. Yusuf and Ibrahim reported that 56.0% of respondents lacked adequate training to manage dengue patients, including identifying warning signs, and recommended to close this gap [24].

In the diagnosis and management domain, respondents failed to recognize the importance of NS1 antigen as an early marker of dengue infection. This might indicate a lack of training, experience of managing dengue patients or a lack of diagnostic test kits for detecting NS1 antigen in health facilities as has been reported in other countries [24]. IgM would be negative during the febrile phase (it may be positive at or after defervescence), while NS1 antigen becomes detectable as early as day one of onset of the disease [1]. Ruberto et al. [25] reported that less than half of the healthcare providers were choosing the right diagnostic tools in the U.S. Respondents also performed poorly on knowledge regarding the detection of plasma leakage using Chest X-rays. This could be in part due to unavailability of X-ray machines in many facilities like BHUs, where they depend on simple blood test parameters, noting that 47.7% of the respondents from hospitals reported using Chest X-ray, while only 11.5% from BHUs reported the use of Chest X-ray for detecting plasma leakage. The feasibility of cheaper, portable and a point-of-care ultrasound machines may be considered to detect plasma leakage and other lifesaving diagnoses in remote areas without X-ray facilities [26].

Respondents also overlooked the importance of recognizing co-morbidities that require admission for patients suspected of having dengue. A similar pattern was also observed in Ecuador, where only 22.0% of healthcare providers were reported to be monitoring dengue patients having co-morbidities for hospital admission [12]. Patients with significant co-morbidities should be closely monitored before they can be sent back home regardless of the severity of the disease [14]. Respondents also had a poor understanding of elevated haematocrit as an indication for fluid replacement during the course of dengue illness. A similar finding was reported in Puerto Rico, where 30.0% of respondents correctly identified elevated haematocrit after an initial trial of intravenous crystalloids [23].

Only 47.4% of participants knew that aggregate cases, whether suspected or confirmed (based on the WHO case definition) have to be reported weekly. This suggests the need to evaluate and reinforce training of physicians on surveillance and reporting. Training on national disease surveillance and reporting is conducted every year by the Ministry of Health to sensitize healthcare workers, including physicians. There is a need to review the existing strategy of training healthcare workers on the surveillance system in the country to improve reporting.

The high proportion of respondents who had poor knowledge of transmission and clinical course might be due to the lack of experience in dengue case management by the respondents. This is evident with 44% of the respondents having never seen dengue in their professional career. In Bhutan, dengue transmission only occurred in Chukha district (Fig 3) from 2004 to 2012 [27]. Eventually, it spread to eight new districts by the end of 2018, and a further 10 districts in 2019 [28]. Prior to 2019 dengue epidemic, training on dengue management was mostly conducted among clinicians and healthcare workers in the dengue-endemic districts. Since 2019, dengue is reported from 19 districts in Bhutan. Therefore, it is an opportune time to train and provide refresher courses to all clinicians and health works of Bhutan.

Overall, knowledge on transmission, clinical course, diagnosis and management, and surveillance and prevention of dengue was higher for medical doctors, physicians working in hospitals and healthcare workers who had experience in managing dengue cases in their clinical practice. In hospitals, most of the physicians are medical doctors, which might have led to increased performance as compared to those working in BHUs, which are mostly staffed by HAs/COs. Variation in performance between the type of profession may be related to their educational qualifications and responsibilities. A study in Ethiopia has shown that physicians were 39 times more likely to have a high level of knowledge on dengue prevention than nurses [24]. The same study also demonstrated that healthcare workers who worked in referral hospital settings were 75 times more likely to have a high level of knowledge on dengue prevention when compared to those who worked in health centres [24]. Additionally, physicians with past dengue clinical experience had better knowledge than dengue-inexperienced physicians [14], and findings of significant differences in knowledge of dengue among healthcare workers in different clinical settings have been reported in other regions [6, 15]. This emphasizes the need to prioritize specific health centres and novice health professionals in dengue case management training and continuing medical education to reduce morbidity and mortality due to dengue.

This study has some limitations worth mentioning. The response rate was low despite our endeavours to increase it by extending the duration of the survey for collecting the data by more than two months. Firstly, respondents located in remote or hard-to-reach areas with limited internet connectivity may not have accessed the survey despite a follow-up reminder to participate in the survey. Secondly, due to limited health workforce, respondents might have other competing priorities like patient care and health promotion activities at the time of the survey and had to forego their participation. Thirdly, as the number of respondents were higher in the southern region, we speculate that respondents working in the dengue-endemic districts including Chukha, Samtse, Sarpang, Zhemgang, Pemagatshel and Samdrup Jongkhar were more likely to take part in a survey as compared to those working in non-endemic areas due to the familiarity with the disease. High response rates in Thimphu can be attributed to the national referral hospital, where severe dengue patients are referred from other districts. However, this survey does include respondents from all 20 districts, thus covering the whole country.

## Conclusion

The study reveals a critical gap and an urgent need to strengthen the knowledge of dengue management and clinical practices among physicians in Bhutan. Emphasis has to be given to educating physicians on transmission, clinical manifestations, early diagnosis and appropriate management of dengue patients. In addition, strategies should be put in place to enhance the physician’s awareness of surveillance and reporting requirements. Exploratory studies such as focus group discussions with healthcare workers in both hospitals and BHUs or PHCs is suggested to understand critical gaps in dengue management.

## Supporting information

S1 Table Comparison of responses across four domains by the characteristics of the study respondents, Bhutan, 2019–2020. (DOCX)

S1 Appendix Research instrument. (DOCX)

S2 Appendix Dataset. (XLSX)
