# Supplementary material for: Medical practitioner’s knowledge on dengue management and clinical practices in Bhutan
Source: PLoS One. 2021 Jul 16;16(7):e0254369. doi: 10.1371/journal.pone.0254369 (PMC8284660; doi:10.1371/journal.pone.0254369)
Supplement: S1 Table — (DOCX) [file pone.0254369.s001.docx]

| Variables | Response selected | Domain 1 (n=9) | | Domain 2 (19) | | Domain 3 (n=19) | | Domain 4 (n=8) | |
| --- | --- | --- | --- | --- | --- | --- | --- | --- | --- |
|  |  | Median (IQR) | p-value | Median (IQR) | p-value | Median (IQR) | p-value | Median (IQR) | p-value |
| Age groups (years) | <= 29 | 5 (4 - 6) | 0.778⃰⃰⃰ | 9 (6 - 14) | 0.607⃰ | 9 (7 - 15) | 0.879⃰ | 6 (5 - 7) | 0.234⃰ |
|  | 30-39 | 5 (4 - 6) |  | 10.5 (7 - 15) |  | 11.5 (6 - 16) |  | 7 (4 - 7) |  |
|  | >=40 | 5 (4 - 6.5) |  | 9 (7 - 11) |  | 10.5 (8 - 13) |  | 5.5 (4 - 6) |  |
|  |  |  |  |  |  |  |  |  |  |
| Sex | Male | 5 (4 - 6) | 0.817^#^ | 10 (7 - 15) | 0.558^#^ | 11.5 (7 - 15) | 0.594^#^ | 6 (4 - 7) | 0.522^#^ |
|  | Female | 5 (4 - 6) |  | 9 (5 - 14) |  | 9 (7 - 13) |  | 6 (5 - 7) |  |
|  |  |  |  |  |  |  |  |  |  |
| Medical role | HA/COs | 5 (4 - 6) | **0.024**^#^ | 8.5 (5 - 10) | **< 0.001**^#^ | 8 (6 - 10.5) | **< 0.001**^#^ | 6 (4 - 7) | **0.002**^#^ |
|  | Doctor | 6 (4 - 7) |  | 14 (10 - 16) |  | 15 (13 - 17) |  | 7 (6 - 7) |  |
|  |  |  |  |  |  |  |  |  |  |
| Medical experience (years) | <=5 | 5 (4 - 6) | 0.319^#^ | 10 (5 - 15) | 0.902^#^ | 12 (7 - 15) | 0.447^#^ | 6 (4 - 7) | 0.556^#^ |
|  | > 5 | 5 ( 4 - 7) |  | 10 (7 - 13) |  | 9 (7 - 13) |  | 6 (5 - 7) |  |
|  |  |  |  |  |  |  |  |  |  |
| Facility type | Hospital | 6 (5 - 7) | **<0.001**^#^ | 13 (9.5 - 15) | **< 0.001**^#^ | 15 (12 - 16) | **< 0.001**^#^ | 6 (5 - 7) | 0.120^#^ |
|  | BHU | 4.5 (4 - 5) |  | 8 (5 - 10) |  | 8 (5.5 - 10.5) |  | 6 (4 - 7) |  |
|  |  |  |  |  |  |  |  |  |  |
| Ever diagnosed dengue | Yes | 5.5 (4 - 7) | **0.012**^#^ | 12.5 (9 - 15) | **< 0.001**^#^ | 14 (12 - 16) | **< 0.001**^#^ | 7 (5 - 7) | **0.001**^#^ |
|  | No | 5 (4 - 5) |  | 7 (5 - 10) |  | 7 (5 - 9) |  | 5 (4 - 6) |  |

⃰ Kruskal-Wallis test; ^#^Man-Whitney U test
