# Supplementary material for: Medical practitioner’s knowledge on dengue management and clinical practices in Bhutan
Source: PLoS One. 2021 Jul 16;16(7):e0254369. doi: 10.1371/journal.pone.0254369 (PMC8284660; doi:10.1371/journal.pone.0254369)
Supplement: S1 Appendix — (DOCX) [file pone.0254369.s002.docx]

**Research Instrument**

As a part of our efforts to understand the knowledge and clinical practices among medical practitioners on dengue, we are conducting this survey. The survey has 40 questions and it might take 15-20 minutes to complete. Your time is highly valuable and thank you for the participation

**Part I: General Information** Date of survey: ___________

| 1. May I know the Dzongkhag under which your health center is located? | 1. Bumthang  2. Chukha  3. Dagana  4. Gasa  5. Haa  6. Lhuentse  7. Mongar  8. Paro  9. Pemagatshel  10. Punakha  11. S/Jongkhar  12. Samtse  13. Sarpang  14. Thimphu  15. Trashigang  16. Trongsa  17. Tsirang  18. Wangdiphodran  19. T/Yangtse  20. Zhemgang |  |
| --- | --- | --- |
| 2. Which level of healthcare do you practice your medicine? | 1. National  2. Regional  3. General hospital  4. Basic Health Unit (BHU) |  |
| 3. What is your age (in completed year) |  |  |
| 4. What is your gender? | 1. Male  2. Female  3. Others |  |
| 5. What is the title/position of your profession? | 1. Health Assistant  2. Clinical Officer  3. Doctor |  |
| 6. Which of the following best describes your area of speciality, or what is your current working place of speciality? | 1. Community Health  2. General medicine  3. Paediatric  4. Emergency  5. Intensive  6. Laboratory  7. Others, specify |  |
| 7. How many years have you been in this profession?(from the start of career till date): | | |

**Part II: Knowledge and clinical practices**

**a. Dengue transmission**

8. How is dengue transmitted?

1. Bite of *Anopheles* mosquitoes
2. Bite of *Aedes* mosquitoes
3. Bite of *Culex* mosuitoes
4. Bite of *ticks/mites*

9. At what time of the day is people more likely to get infected by dengue? (Multiple choice question)

1. Morning
2. Afternoon
3. Evening
4. Night

10. When mosquito feeds dengue case, which of the following is true?

1. No infection of the mosquito, if the dengue virus concentration in the blood is too low
2. The mosquito becomes infected and can infect others when it feeds next
3. The mosquito will live for another 2-3 months
4. Female mosquitoes will spread the dengue virus to other mosquitoes when they mate

11. So far, which of the following dengue virus (DENV) serotypes are found in Bhutan? (Multiple choice question)

1. DENV-1
2. DENV-2
3. DENV-3
4. DENV-4

**b. Clinical presentation and course**

12. How many cases of dengue have you personally diagnosed during your career?

1. 0
2. 5 or less
3. 6–10
4. 11–20
5. >21 cases

13. After being bitten by dengue infected mosquitoes, how long does it take to develop dengue illness?

1. Less than 3 days after exposure
2. Between 3-10 days after exposure
3. As long as 3 weeks after exposure
4. Anytime in the month after exposure

14. How should a clinician identify a patient with dengue at the time of presentation? (Multiple choice question)

1. Take a fever history to identify any reported fever within the past week
2. Only consider dengue if the patient is febrile on presentation
3. Perform a complete blood count to look for hemoconcentration, leukopenia, or thrombocytopenia
4. Ask about headache, retro-orbital pain, joint pain, rash, and muscle aches
5. Ask about vomiting, abdominal pain, bleeding, and lethargy
6. Only consider dengue if thrombocytopenia is present

15. Identify warning signs of dengue fever or severe dengue from the following list (multiple choice question)

| 1. Fever | 2. Persistent vomiting | 3. Muscle pain |
| --- | --- | --- |
| 4. Dyspnoea | 5. Giddiness | 6. Constipation |
| 7. Retro-orbital pain | 8. Lethargy | 9. Diarrhoea |
| 10. Persistent cough | 11. Bleeding | 12. Abdominal pain |

16. A 10-year-old female patient presents to you with 2 days history of fever, headache, joint pain, vomiting, mucosal bleeding, lethargic and liver enlargement (>2cm). Based on the latest WHO guideline, this patient is classified as:

1. Dengue fever
2. Dengue haemorrhagic fever
3. Dengue with warning signs
4. Severe dengue

17. A severe dengue should be considered if the patient from an area of dengue risk presenting with fever of 2-7 days plus any of the following features (Multiple choice question)

1. An evidence of plasma leakage
2. A significant bleeding
3. Altered level of consciousness (lethargy or restlessness, coma)
4. A severe gastrointestinal involvement (persistent vomiting, increasing or intense abdominal pain, jaundice)
5. [Guillain–Barre syndrome](https://bmcresnotes.biomedcentral.com/articles/10.1186/s13104-015-1672-0)

6. Severe organ impairment (acute renal/liver failure, encephalitis, cardiomyopathy)

18. Which of the following is the best early indicator of shock?

1. Hypotension
2. Bradycardia
3. Mental status changes
4. Tachycardia in the absence of fever or delayed capillary refill

19. When do you expect a patient to develop clinically significant plasma leakage?

1. During the first 3 days of illness while the patients is febrile
2. Usually on days 3–7 of illness, around the time of defervescence
3. Following a rapid increase in platelet count and progressive leucocytosis
4. During the recovery phase

20. When do you expect a patient with dengue to develop thrombocytopenia?

1. In the first 3 days of the illness, while the patient is febrile
2. During the recovery phase
3. Thrombocytopenia is not seen in dengue
4. Following progressive leukopenia, around the time of defervescence

**c. Diagnosis**

21. What is true about testing for dengue virus? (Multiple choice question)

1. Absence of anti-dengue IgM antibodies rules out acute infection
2. Anti-dengue IgM antibodies are first detectable in most patients on days 3–5 after illness onset
3. No test exists to diagnose acute dengue virus infection before antibody production
4. Anti-dengue IgG antibodies detected in days 1– 3 after onset can be used to diagnose an acute dengue virus infection

22. How can clinically significant plasma leakage be detected in a suspected dengue patient? (Multiple choice question)

1. Decreasing haematocrit
2. Increasing hematocrit above 20% of baseline
3. Decreasing platelet count
4. Chest X-ray with lateral decubitus view to look for pleural effusion

**d. Clinical management**

23. A 6-year-old boy patient presents to your office with a few days of fever and a distended, painful abdomen. Her mother states that she has been less active over the past three days. It is currently April and you have seen six patients in the past three weeks with dengue infections. The best course of action in managing this patient is to:

1. Order dengue lab tests and ask the patient to return to your office in 24 hours
2. Order dengue lab tests and admit the patient to the hospital for 24 hours of observation
3. Order dengue lab tests and admit the patient to the Intensive Care Unit for close monitoring and access to emergency care

24. Select an appropriate treatment you could use to treat dengue fever (Multiple choice question)

| 1. Aspirin | 2. Opioids | 3. Paracetamol |
| --- | --- | --- |
| 4. Oral hydration | 5. Antiviral treatment | 6. Blood transfusion |

25. Which of the following are the correct criteria for sending a suspected dengue patient home? (Multiple choice question)

1. Passing urine at least once every 6 hours
2. Has abdominal pain but no persistent vomiting
3. Does not have coexisting conditions such as diabetes
4. Has a pulse pressure less than 20
5. Does not have any warning signs
6. No hemoconcentration

26. When and under what circumstances should you tell a patient with suspected dengue to return to the clinic or present to local emergency department? (Multiple choice question)

1. Persistent vomiting
2. Drowsiness or lethargy
3. Hematemesis
4. High fever (> 38°C)

27. When should intravenous (IV) crystalloids (i.e., Ringer’s lactate, normal saline) be given to suspected dengue patients? (Multiple choice question)

1. All cases regardless of clinical evidence of shock or hemoconcentration
2. Hypotension, as initial fluid replacement therapy
3. Significant clinical bleeding, as initial fluid replacement therapy
4. Low hematocrit and persistent shock after initial IV fluids given
5. High hematocrit, as initial fluid replacement therapy
6. Tachycardia, delayed capillary refill, or low urine output as initial IV fluid therapy

28. When should blood transfusions (whole blood or packed red blood cells) be given for a patient suspected of having dengue? (Multiple choice question)

1. Hypotension as a presenting symptom in a suspected dengue patient
2. Low platelet count without bleeding, shock, or hypotension
3. Significant clinical bleeding
4. Low hematocrit and persistent shock after a trial of IV crystalloids and/or colloids
5. Blood transfusions should never be given

**e. Surveillance and prevention of dengue**

29. Which of the following statements is correct regarding the reporting of dengue in Bhutan? (Multiple choice question)

1. Dengue is not a notifiable disease
2. Dengue reporting should occur with laboratory confirmed cases only
3. All suspected and confirmed cases should be reported within 1 week
4. Severe dengue should be reported immediately after it is detected in the healthcenter

30. Which variables are required to report dengue in the surveillance system (NEWARS)? (Multiple choice question)

1. Age
2. Sex
3. Type of case (whether case or death)
4. Signs and symptoms
5. Travel history

**f. Prevention messages**

31. Dengue vaccine is recommended for those aged 9 years and above in an area with

1. ≤49% prevalence 2. 50-59% prevalence

3. 60-69 %prevalence 4. ≥ 70% prevalence

32. What prevention messages should be given to patients to avoid dengue virus infection? (Multiple choice question)

1. Travelers to endemic areas should be offered vaccination
2. Use mosquito repellent and wear long sleeves and pants for protection
3. Avoid mosquitos during the peak feeding time at night
4. If someone in the household is sick with dengue, take precautions so that a mosquito does not bite the infected person and then bite others in the household.

THANK YOU FOR THE PARTICIPATION!
